# CdSe/ZnS quantum dots exhibited nephrotoxicity through mediating oxidative damage and inflammatory response

**DOI:** 10.18632/aging.103774

**Published:** 2020-11-16

**Authors:** Xiuli Li, Huiwu Zhang, Fuyun Sun

**Affiliations:** 1 Department of Nephrology, Cangzhou Central Hospital, Cangzhou, Hebei Province, China; 2 Pediatrics Department, Cangzhou Central Hospital, Cangzhou, Hebei Province, China

**Keywords:** nephrotoxicity, CdSe/ZnS quantum dots, oxidative damage, inflammatory response, NRF2/Keap1 pathway

## Abstract

Objective: This study aimed to the evaluate the nephrotoxicity of CdSe/ZnS QDs *in vitro* and *vivo*, as well as investigate the underlying toxicity mechanisms.

Results: *In vitro* experiments showed that compared with control cells, CdSe/ZnS QDs treatment significantly inhibited cell viability and promoted cell apoptosis in dose-dependent manner in NRK cells. Notably, CdSe/ZnS QDs treatment increased the contents of MDA and ROS, and decreased the activities of SOD, CAT and GSH-Px; however, the co-treatment of NAC and QDs relieved the oxidative damage of NRK cells. Moreover, *in vivo* experiments also revealed that CdSe/ZnS QDs treatment obviously increased kidney weight coefficient, damaged the kidney function, as well as induced inflammatory response and inhibited the activation of NRF2/Keap1 pathway in kidney tissues of mice.

Conclusions: CdSe/ZnS QDs exhibited obvious nephrotoxicity by mediating oxidative damage and inflammatory response *in vitro* and *in vivo* via NRF2/Keap1 pathway.

Methods: The characterization of CdSe/ZnS QDs was analyzed by transmission electron microscope, emission spectrum scanning, and dynamic light scattering. Rat kidney cells (NRK) were exposed to different doses of CdSe/ZnS QDs with or without N-acetylcysteine (NAC, antioxidant). Then, cellular uptake of CdSe/ZnS QDs was detected, and *in vitro* cytotoxicity was evaluated by MTT assay and TUNEL assay.

## INTRODUCTION

In recent years, nanomaterials have been reported to be promising functional materials and display great application values in various fields, including materials, information, energy and biomedicine [1]. However, the attendant potential risks, such as human exposure and environmental contamination, have limited the expanding commercial applications of several nanomaterials [2]. Notably, quantum dots (QDs) as one class of nanomaterials have attracted tremendous attentions due to its unique structural and mechanical properties [3]. QDs are fluorescent semiconductor nanocrystals consisting of group II-VI or III-V elements, characterized with the property of narrow and sharp emission spectra as well as broad excitation spectra [4]. Currently, QDs have been extensively applied in biomedical fields, including cellular imaging [5] and drug delivery [6]. Especially, QDs facilitate to uncover cellular processes by imaging, and are used as a tool of immunodetection during basic biomedical research [7, 8]. In addition, QDs can be applied for the development of highly sensitive cancer diagnostic system [9, 10]. Unfortunately, despite the biological effects of QDs have been proved, the toxicity and safety remain serious issues [11]. The most popular QDs, such as CdS, CdTe, and CdSe, are cadmium (Cd)-based QDs, while Cd possesses high toxicity and carcinogenicity to liver [12, 13]. It has been revealed that QDs can be oxidized after entering into cells, which lead to the release of Cd into cell environment [14]. However, few studies have investigated the nephrotoxicity of CdSe/ZnS QDs.

In the present study, the characterization of CdSe/ZnS QDs was first measured. Afterwards, the nephrotoxicity of CdSe/ZnS QDs was evaluated *in vitro* and *in vivo* experiments, and the potential mechanism was further investigated.

## RESULTS

### Characterization of CdSe/ZnS QDs

**Figure 1:**
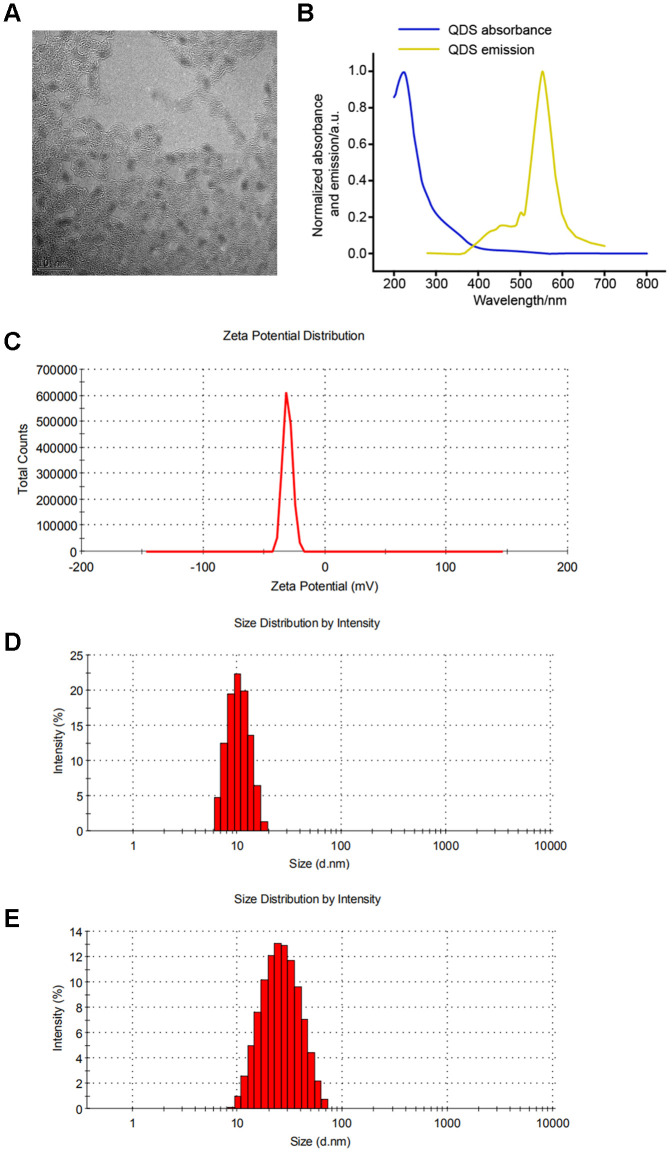
**Characterization of CdSe/ZnS quantum dots (QDs).** (**A**) The morphologies of CdSe/ZnS QDs by transmission electron microscopy. (**B**) The emission spectrum scanning of CdSe/ZnS QDs. (**C**) The zeta potential analyses and dynamic light scattering plots of CdSe/ZnS QDs cultured in DMEM for 0 (**D**) and 24 h (**E**).

As shown in Figure 1A, TEM showed that solubilized CdSe/ZnS QDs was monodisperse with the small size distribution (Figure 1A). Accordingly, CdSe/ZnS QDs by emission spectrum scanning revealed the fluorescence maximum at about 550 nm (Figure 1B). In addition, DLS showed the excellent homogeneity with the similar size of around 10 nm at 0 and 24 h (Figure 1C-E), which suggested the stability of CdSe/ZnS QDs.

### Effect of CdSe/ZnS QDs on cytotoxicity in NRK cells

**Figure 2:**
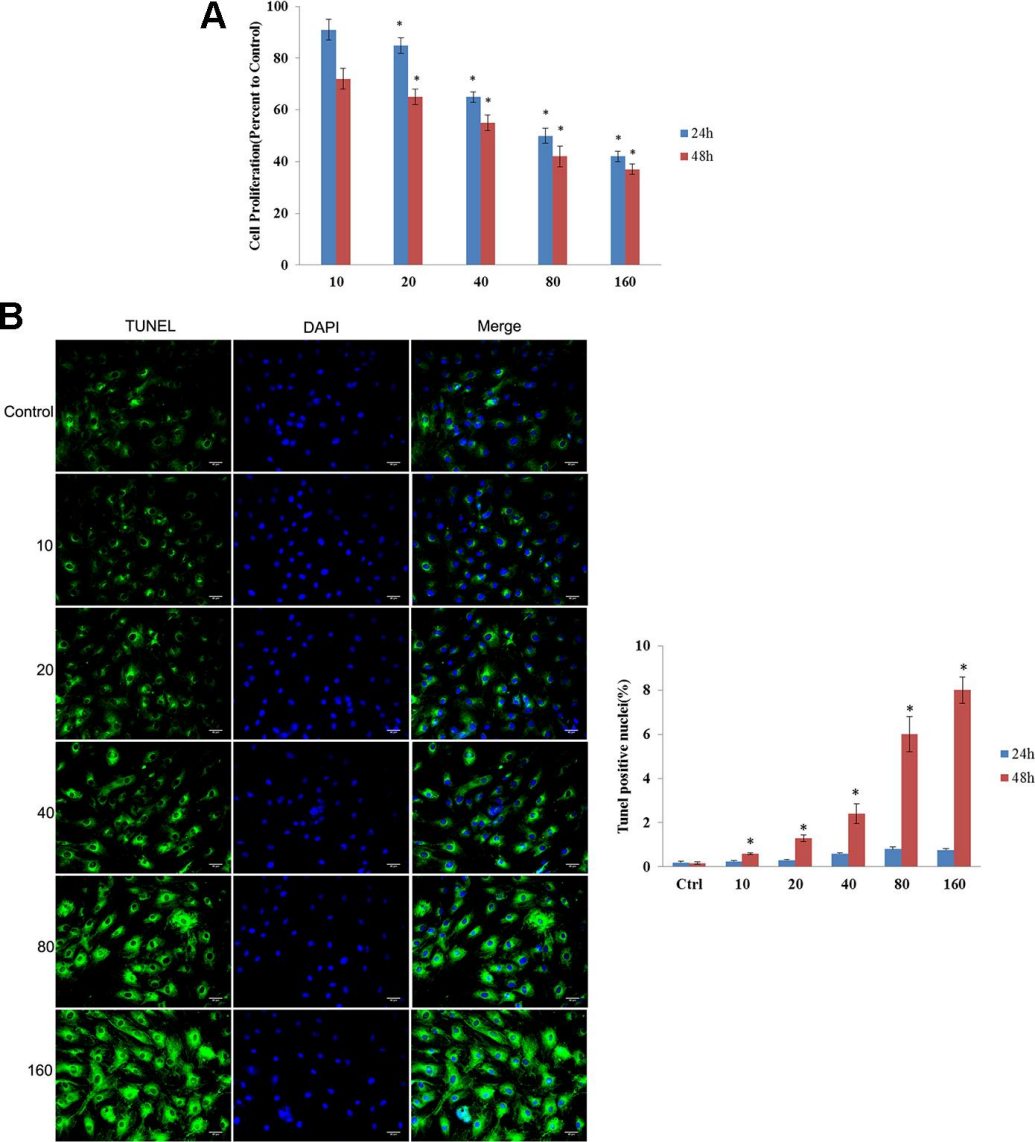
**CdSe/ZnS QDs exhibited toxicity to NRK cells.** (**A**) Cell viability of NRK cells treated with different doses of CdSe/ZnS QDs at 24 and 48 h using MTT assay. (**B**) Cell apoptosis of NRK cells treated with different doses of CdSe/ZnS QDs at 24 and 48 h using TUNEL staining. *P < 0.05 vs. 10 nM group or control group.

Cytotoxicity of CdSe/ZnS QDs was evaluated by MTT assay and TUNEL assay. MTT assay showed that CdSe/ZnS QDs treatment significantly inhibited cell viability of NRK cells in a dose-dependent manner both at 24 and 48 h (Figure 2A). Consistently, compared control cells, TUNEL-stained cells were obviously increased in a dose-dependent manner after NRK cells treated with CdSe/ZnS QDs both at 24 and 48 h (Figure 2B). These results suggested CdSe/ZnS QDs exhibited toxicity to NRK cells.

### Cellular uptake of CdSe/ZnS QDs in NRK cells

**Figure 3:**
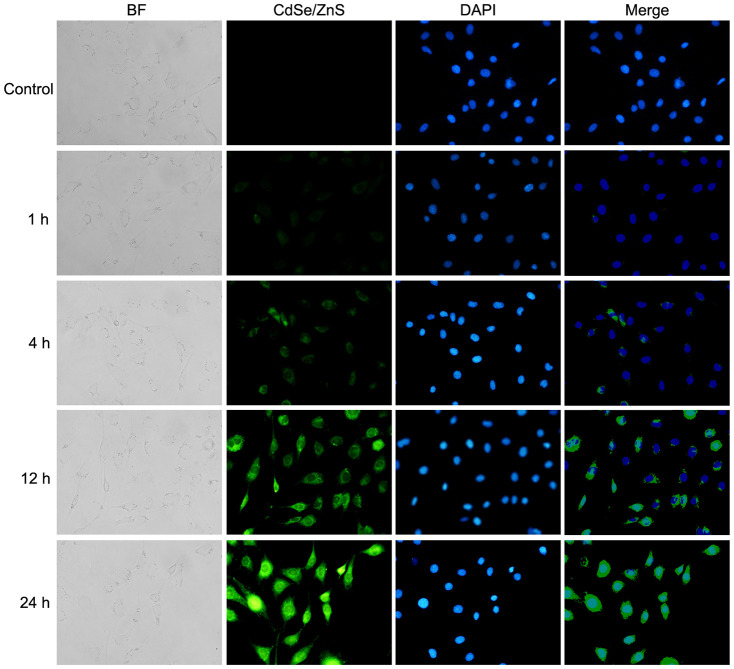
**Cellular uptake of CdSe/ZnS QDs in NRK cells.** Confocal images of NRK cells treated with 40 nM of CdSe/ZnS QDs for 0, 1, 4, 12, and 24 h.

Based on confocal imaging analysis, we found the enhanced fluorescence intensity when cells were treated with CdSe/ZnS QDs compared with control cells; meanwhile, the fluorescence intensity was increased in time-dependent manner (Figure 3). These results indicated the high cellular uptake of CdSe/ZnS QDs in NRK cells.

### Effect of CdSe/ZnS QDs on oxidative damage in NRK cells

**Figure 4:**
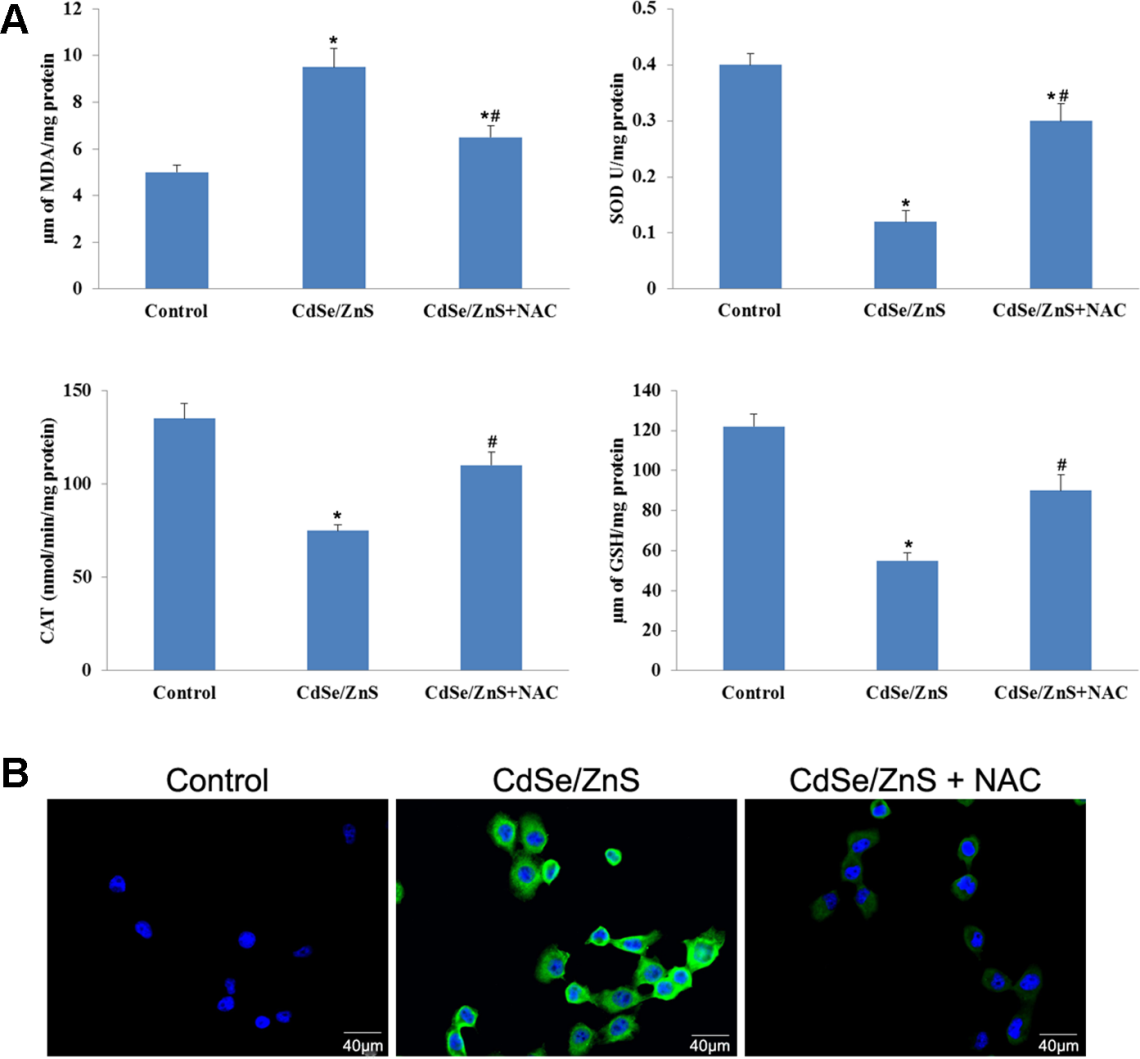
**CdSe/ZnS QDs induced oxidative damage in NRK cells.** (**A**) Measurements of several oxidative damage indicators, including malondialdehyde (MDA), catalase (CAT), superoxide dismutase (SOD), and glutathione peroxidase (GSH-Px), in NRK cells treated with CdSe/ZnS QDs and/or N-acetylcysteine (NAC, antioxidant) using the commercial kits. (**B**) Reactive oxygen species (ROS) content in NRK cells treated with CdSe/ZnS QDs and/or NAC using the commercial kit. *P < 0.05 vs. control group; ^#^P < 0.05 vs. CdSe/ZnS group.

Compared with control cells, CdSe/ZnS QDs treatment significantly increased MDA content and ROS content, while decreased the activities of SOD, CAT and GSH-Px in NRK cells (p < 0.05, Figure 4A, 4B). However, the co-treatment of NAC and CdSe/ZnS QDs exhibited lower contents of MDA and ROS, as well as elevated activities of SOD, CAT, and GSH-Px compared with the single CdSe/ZnS QDs treatment (p < 0.05, Figure 4A, 4B). These data suggested that CdSe/ZnS QDs induced oxidative damage in NRK cells.

### Effect of CdSe/ZnS QDs on kidney function *in vivo*

**Figure 5:**
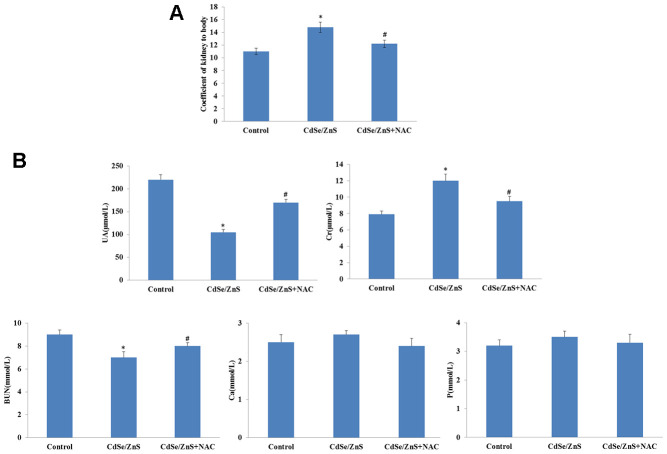
**CdSe/ZnS QDs caused kidney disorder in mice.** (**A**) The kidney weight coefficient of mice after CdSe/ZnS QDs and/or N-acetylcysteine (NAC, antioxidant) injection. (**B**) Measurements of biochemical indicators of renal function, including uric acid (UA), urea nitrogen (BUN), creatine (Cr), calcium (Ca) and phosphorus (P), using clinical automatic chemistry analyzer. *P < 0.05 vs. control group; ^#^P < 0.05 vs. CdSe/ZnS group.

The toxicity of CdSe/ZnS QDs was explored in kidney tissues of mice. The kidney weight coefficient was significantly increased after CdSe/ZnS QDs treatment, while the addition of NAC reduced the kidney weight coefficient (Figure 5A). In addition, the biochemical indicators of renal function were measured, and the results revealed that CdSe/ZnS QDs treatment remarkably inhibited the contents of UA, Cr, and BUN (p < 0.05), as well as reduced the contents of Ca and P while without significant difference (Figure 5B). However, the addition of NAC partly reversed the contents of UA, Cr, BUN, Ca and P induced by CdSe/ZnS QDs (Figure 5B).

### Effect of CdSe/ZnS QDs on inflammatory response *in vivo*

**Figure 6:**
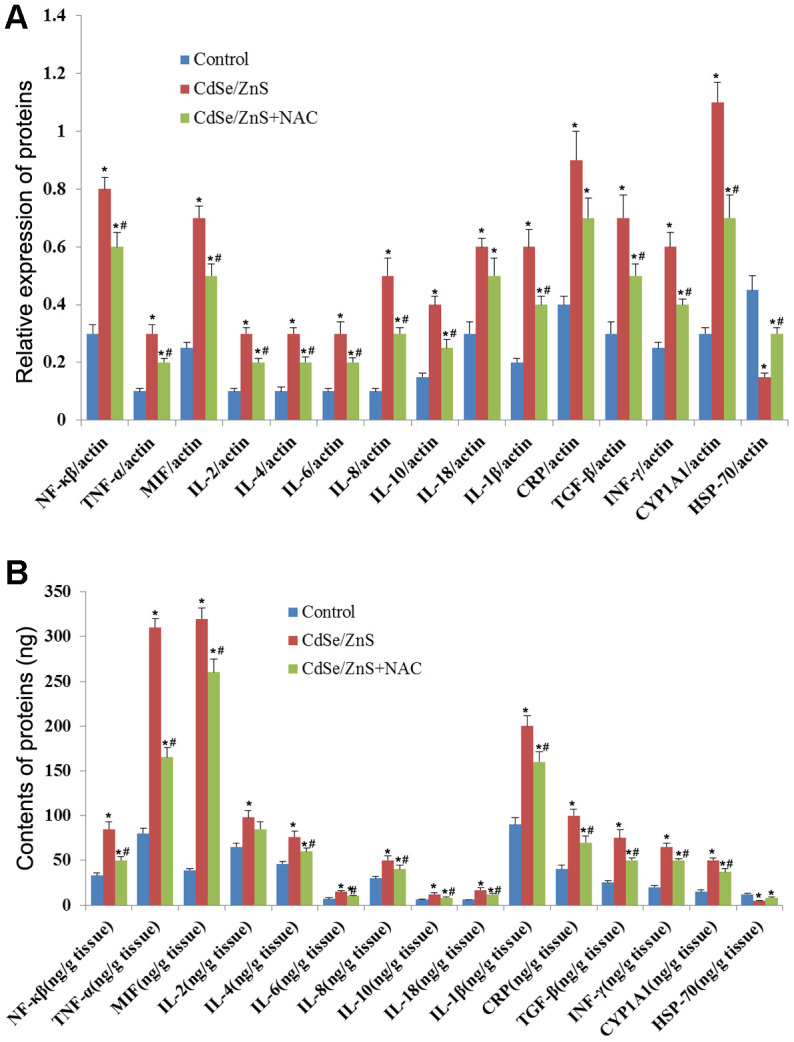
**CdSe/ZnS QDs induced inflammatory response in mice.** (**A**) The mRNA levels of inflammatory cytokines, including nuclear transcription factor-kB (NF-kB), tumor necrosis factor α (TNF-α), macrophage migration inhibitory factor (MIF), interleukin-2 (IL-2), IL-4, IL-6, IL-8, IL-10, IL-18, IL-1β, C-reactive protein (CRP), transforming growth factor (TGF-β), interferon (INF-γ), cytochrome P450 1A (CYP 1A1) and heat shock protein 70 (HSP70), in kidney tissues of mice after CdSe/ZnS QDs and/or N-acetylcysteine (NAC, antioxidant) injection by qRT-PCR. (**B**) The protein levels of these inflammatory cytokines by enzyme-linked immunosorbent assay. *P < 0.05 vs. control group; ^#^P < 0.05 vs. CdSe/ZnS group.

We also detected the expression of inflammatory cytokines to evaluate the toxicity of CdSe/ZnS QDs to kidney tissues of mice. qRT-PCR revealed that CdSe/ZnS QDs treatment conspicuously increased the mRNA levels of proinflammatory cytokines, including IL-1β, IL-2, IL-4, IL-18, IL-10, IL-8, IL-6, INF-γ, TGF-β, CRP, MIF, TNF-α, NF-kB, and CYP1A1, while decreased the mRNA level of HSP70 (p < 0.05, Figure 6A). However, the co-treatment of NAC and CdSe/ZnS QDs exhibited lower mRNA levels of proinflammatory cytokines, including IL-1β, IL-2, IL-4, IL-18, IL-10, IL-8, IL-6, INF-γ, TGF-β, CRP, MIF, TNF-α, NF-kB, and CYP1A1, as well as higher mRNA level of HSP70 compared with the single CdSe/ZnS QDs treatment (p < 0.05, Figure 6A). Similarly, ELISA results revealed that the protein expression of these inflammatory cytokines emerged the consistent expression trend compared with the results of qRT-PCR (Figure 6B).

### Effect of CdSe/ZnS QDs on Nrf2/KEAP1 pathway *in vivo*

**Figure 7:**
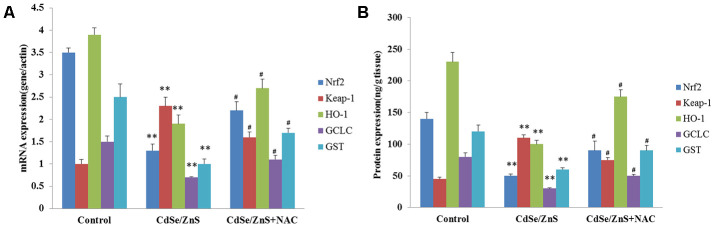
**CdSe/ZnS QDs inhibited the activation of Nrf2/Keap1 pathway *in vivo*.** (**A**) The mRNA levels of Nrf2/KEAP1 pathway related genes, including Nrf2, Kelch-like ECH-associated protein 1 (Keap1), heme oxygenase (HO-1), glutamate cysteine ligase catalytic subunit (GCLC), glutathione S-transferase (GST), in kidney tissues of mice after CdSe/ZnS QDs and/or N-acetylcysteine (NAC, antioxidant) injection by qRT-PCR. (**B**) The protein levels of these Nrf2/KEAP1 pathway related proteins by enzyme-linked immunosorbent assay. *P < 0.05 vs. control group; ^#^P < 0.05 vs. CdSe/ZnS group.

It is well-known that oxidative damage was associated with NRF2/KEAP1 pathway, thus, the key genes related to NRF2/KEAP1 pathway were detected. RT-PCR analysis showed that CdSe/ZnS QDs treatment remarkably decreased the mRNA levels of NRF2, HO-1, GCLC, and GST, and increased the mRNA level of Keap1 compared with control cells (p < 0.01, Figure 7A), while the addition of NAC partly reversed the mRNA levels of NRF2, HO-1, GCLC, GST, and Keap1 compared to cells treated with CdSe/ZnS QDs (p < 0.05, Figure 7A). In addition, ELISA also revealed that compared with control cells, the protein levels of NRF2, HO-1, GCLC, and GST were obviously reduced, and the protein level of Keap1 was elevated (p < 0.01, Figure 7B). However, the co-treatment of NAC and CdSe/ZnS QDs promoted the protein levels of NRF2, HO-1, GCLC, and GST as well as suppressed the protein level of Keap1 compared with the single CdSe/ZnS QDs treatment in kidney tissues of mice (p < 0.05, Figure 7B).

## DISCUSSION

An exceptional array of physicochemical properties have attracted the attention of many researchers to explore the biomedical applications of QDs, while the following security concerns are raised. To date, the nephrotoxicity of CdSe/ZnS QDs has not been well investigated, and the underlying toxicity mechanisms also should be further understood. In the current study, *in vitro* experiments showed that compared with control cells, CdSe/ZnS QDs treatment significantly inhibited cell viability and promoted cell apoptosis with the increasing dose in NRK cells. Notably, CdSe/ZnS QDs treatment increased the contents of MDA and ROS, decreased the activities of SOD, CAT and GSH-Px in NRK cells; however, the co-treatment of NAC and QDs relieved the oxidative damage of NRK cells. Moreover, *in vivo* experiments also revealed that CdSe/ZnS QDs treatment obviously increased kidney weight coefficient, damaged the kidney function, as well as induced inflammatory response in kidney tissues of mice, while the addition of NAC alleviated renal injury and inflammatory response. Furthermore, NRF2/KEAP1 pathway was inhibited by CdSe/ZnS QDs treatment, which might be associated with the nephrotoxicity of CdSe/ZnS QDs.

Considerable evidence has demonstrated that CdSe/ZnS QDs can be widely used for various biological and biomedical applications due to its unique physiochemical properties. However, the toxicity and safety of CdSe/ZnS QDs on the human health are still considered as key factors in biomedical applications. Cytotoxicity studies of CdSe/ZnS QDs have been conducted in several *in vitro* experiments. Peng *et al.* [15] have demonstrated that CdSe/ZnS QDs are able to suppress cell viability in HepG2 cells, while CdSe/ZnS QDs with a dose > 100 nM exerts significant cytotoxicity. Also, Manshian *et al.* [16] have evaluated the toxicity of CdSe/ZnS QDs in different cell type, including lymphoblastoid (TK6), fibroblast (HFF-1), and epithelial cells (BEAS-2B), and the results exhibits the cytotoxicity of CdSe/ZnS QDs to HFF-1 and TK6 cells, suggesting cell type-dependent variation in the cytotoxicity of CdSe/ZnS QDs. Several studies report that CdSe/ZnS QDs exhibit obvious cytotoxicity in cancer cells, such as pancreatic cancer cells [17] and lung adenocarcinoma cells [18]. Similarly, our study also revealed that CdSe/ZnS QDs treatment inhibited cell viability and promoted cell apoptosis in NRK cells, as well as damaged the kidney function in mice, which suggested the nephrotoxicity of CdSe/ZnS QDs *in vitro* and *in vivo*.

Furthermore, this study investigated the mechanism of the nephrotoxicity of CdSe/ZnS QDs *in vitro* and *in vivo*. Oxidative damage is reported to play major role in cytotoxicity [19]. It is well-known that over-production of ROS can influence the stability of DNA and RNA, and decrease the activities of SOD, CAT and GSH-Px as well as increase MDA content, thereby resulting in cell oxidative damage [20]. Li et al. [21] have reported that CdS QDs treatment significantly increases ROS level, decreases GSH activity, and induces Cd^2+^ release, which is considered to responsible for the cytotoxicity of CdS QDs. Also, Luo *et al.* [22] have shown that Cd-based QDs can induce oxidative stress by increasing the production of ROS in renal adenocarcinoma cells. In this study, we found that CdSe/ZnS QDs increased the contents of MDA and ROS, as well as decreased the activities of SOD, CAT and GSH-Px, as well as lowered the mitochondrial membrane potential in NRK cells. Additionally, inflammatory response is also reported to be closely implicated in nephrotoxicity [23], the anti-inflammation drug development has been focused [24, 25]. Notably, it has been proved that CdSe/ZnS QDs can induce liver inflammation through activating NLRP3 inflammasome [26]. This study revealed that CdSe/ZnS QDs increased the productions of proinflammatory cytokines, including IL-1β, IL-2, IL-4, IL-18, IL-10, IL-8, IL-6, INF-γ, TGF-β, CRP, MIF, TNF-α, NF-kB, and CYP1A1, and decreased the expression of HSP70 in kidney tissues of mice. This study further found that the co-treatment of CdSe/ZnS QDs and NAC alleviated oxidative damage and inflammatory response. It is well-known that NAC as antioxidant has been reported to exert inhibiting effects on oxidative stress and inflammatory response [27, 28]. Consistently, previous studies have demonstrated that NAC exerts inhibiting effect on nephrotoxicity [29–31]. Thus, these results indicated that CdSe/ZnS QDs might induce the nephrotoxicity through inducing oxidative damage and promoting inflammatory response.

Interestingly, our *in vivo* results revealed that CdSe/ZnS treatment inhibited the activation of Nrf2/Keap1 pathway, while which was partly activated by NAC. It has been reported that NRF2/Keap1 signaling pathway plays a critical role during oxidative damage [32]. Under the physiological state, NRF2 is combined with Keap1 in the cytoplasm; however, in the stress state, NRF2 is dissociated from Keap1 and transported into the nucleus, thereby activating the expression of anti-oxidants (such as HO-1, GCLC, and GST) [33]. In addition, the activation of NRF2/Keap1 signaling pathway can promote inflammatory response [34]. Together with these findings, our results indicated that CdSe/ZnS QDs might exert obvious nephrotoxicity via NRF2/Keap1 pathway.

In conclusion, findings from this study revealed that CdSe/ZnS QDs exhibited nephrotoxicity by mediating oxidative stress and inflammatory response *in vitro* and *in vivo*, which was closely related to NRF2/Keap1 pathway.

## MATERIALS AND METHODS

### Characterization of CdSe/ZnS QDs

Water-soluble CdSe/ZnS QDs were purchased from Ocean Nanotech (USA). The characterization of CdSe/ZnS QDs were observed by transmission electron microscope (TEM), emission spectrum scanning, and dynamic light scattering (DLS). Briefly, 8 μM of CdSe/ZnS QDs solution was diluted 20-fold with PBS buffer, and then 10 μL of the sample was dripped onto a carbon-coated copper mesh. After water evaporation, the sample was observed by TEM (Tecnai G2 20 S-TWIN, FEI, Eindhoven, Netherlands). For emission spectrum scanning, 8 μM of CdSe/ZnS QDs solution was diluted to a concentration of 10 nM with PBS buffer. Next, 200 uL of the sample was added into 96-well plate, and detected using M200 multifunctional fluorescence microplate reader at the excitation wavelength of 450 nm and the end wavelength of 750 nm. For DLS, 10 nM of CdSe/ZnS QDs was cultured in DMEM for 0 and 24 h, then detected by Zetasizer Nano Z (Worcestershire, UK) at 0 and 24 h, respectively.

### Cell culture and treatment

Rat kidney cells (NRK) were provided by Shanghai Obio (China), and maintained in RPMI-1640 medium (Gibco) with 10% FBS under 5% CO_2_ and 37°C. NRK cells were exposed to different doses of CdSe/ZnS QDs with or without N-acetylcysteine (NAC, antioxidant, provided by Sigma).

### Thiazolyl blue tetrazolium bromide (MTT) assay

To evaluate the cytotoxicity of CdSe/ZnS QDs, NRK cells were cultured in 96-well plates. On the next day, cells were then incubated with different doses (10, 20, 40, 80, 160 nM) of CdSe/ZnS QDs, respectively, for 24 h and 48 h. Next, MTT (10 μL, Sigma, St Louis, MI, USA) was added to incubate with cells for 4 h, and dimethyl sulfoxide (150 μL, Sigma) was then used to dissolve formazan precipitates. Microplate spectrophotometer was used to evaluate cell viability based on the absorbances at 450 nm.

### The terminal deoxynucleotidyl transferase d UTP nick end labeling (TUNEL) assay

NRK cells were incubated with different doses (10, 20, 40, 80, 160 nM) of CdSe/ZnS QDs for 24 h and 48 h, respectively. Then, cells were fixed and stained with TUNEL according to the producer’s guideline. Followed by TUNEL staining, DAPI was used for staining nuclei. TUNEL-positive cells were photographed and observed by fluorescence microscope (Olympus, Tokyo, Japan).

### Cellular uptake of CdSe/ZnS QDs

Cellular uptake of CdSe/ZnS QDs was evaluated using confocal imaging analysis. Briefly, NRK cells (2.5 × 10^4^) were grown in 24-well plates for 24 h, and then incubated with 40 nM of CdSe/ZnS QDs for 1, 4, 12 and 24 h, respectively. After washed with PBS, cells were stained with Heochst33342 for 10 min, and then observed using confocal fluorescence microscope.

### Measurements of oxidative damage indicators

Oxidative damage indicators, including malondialdehyde (MDA), catalase (CAT), glutathione peroxidase (GSH-Px), superoxide dismutase (SOD) and reactive oxygen species (ROS) were detected in NRK cells treated with 40 nM of CdSe/ZnS QDs and/or 10 μM of NAC for 12 h. MDA content, CAT activity, SOD activity, and GSH-Px activity in cells and tissues were determined using commercial kits (Nanjing Jiancheng, Nanjing, China). Meanwhile, ROS contents in cells and tissues were also determined using DCFH-DA (Beyotime, Beijing, China). The cells were observed under inverted microscope (Olympus, Japan), and fluorescent density was calculated.

### Animal experiments

Approval from the local animal Ethics Committee of the animal laboratory center was obtained prior to experiments. Followed by one week of acclimation, a total of 30 healthy mice were used for the *in vivo* study. Thirty mice were randomly and equally assigned into 3 groups: control group, CdSe/ZnS group, and CdSe/ZnS + NAC group. Mice in the CdSe/ZnS group were injected with 50 mg/kg of CdSe/ZnS QDs via tail vein, while mice in the CdSe/ZnS + NAC group received 50 mg/kg of CdSe/ZnS QDs via intravenous injection and 100 mg/kg/day of NAC via intraperitoneal injection. After 14 days of injection, mice were euthanized, and the kidney tissues were collected. Kidney weight coefficient was measured as following: kidney wet weight (g)/body weight (g)*100. Furthermore, the biochemical indicators of renal function, including calcium (Ca), phosphorus (P), creatine (Cr), urea nitrogen (BUN), and uric acid (UA), were measured by automatic chemistry analyzer (Hitachi, Japan).

### RNA extraction, reverse transcription and real-time quantitative PCR (qRT-PCR)

Total RNA was obtained from kidney tissues by Trizol, and then PrimeScript™ RT reagent Kit (Takara, Dalian, China) was applied to obtain cDNA by reverse transcription of RNA. The qRT-PCR was carried out by the SYBR Premix Ex Taq TM II (Takara). The PCR primers for various inflammatory cytokines, including interleukin-2 (IL-2), IL-4, IL-6, IL-8, IL-10, IL-18, IL-1β, cytochrome P450 1A (CYP 1A1), transforming growth factor (TGF-β), interferon (INF-γ), C-reactive protein (CRP), macrophage migration inhibitory factor (MIF), tumor necrosis factor α (TNF-α), nuclear transcription factor-kB (NF-kB), and heat shock protein 70 (HSP70), as well as Nrf2/KEAP1 pathway related genes, including Nrf2, Kelch-like ECH-associated protein 1 (Keap1), heme oxygenase (HO-1), glutamate cysteine ligase catalytic subunit (GCLC), glutathione S-transferase (GST), and β-actin, are shown in Table 1. β-actin were served as the internal control, and mRNA expression data was evaluated by 2-ΔΔCt method.

**Table 1:** Primers used for the qRT-PCR.

**Gene**	**Primer sequence**
NF-kB	F: 5′-CCTCTACACATAGCGGCTGG-3′
	R: 5′-GCACCTTGGGATGCGTTTTT-3′
TNF-α	F: 5'-CTCCCTCCAGAAAAGACACCAT-3'
	R: 5'-ATCACCCCGAAGTTCAGTAGACAG-3'
MIF	F: 5'-CGGACCGGGTCTACATCAAC-3'
	R: 5'-GAACAGCGGTGCAGGTAAGTG-3'
IL-2	F: 5'-GCCCCAAGGGCTCAAAAATG-3'
	R: 5'-GCGCTTACTTTGTGCTGTCC-3'
IL-4	F: 5'-ACTGCACAGCAGTTCCACAG-3'
	R: 5'-CTCTGGTTGGCTTCCTTCAC-3'
IL-6	F: 5'-CAGAAGGAGTGGCTAAGGACCA-3'
	R: 5'-ACGCACTAGGTTTGCCGAGTAG-3'
IL-8	F: 5'-ATGACTTCCAAGCTGGCCGTGGCT-3'
	R: 5'-TCTCAGCCCTCTTCAAAAACTTCTC-3'
IL-10	F: 5'-GGACTTTAAGGGTTACTTGG-3'
	R: 5'-TCACCCAGGGAATTCAAATG-3'
IL-18	F: 5'-GACCTTCCAGATCGCTTCCTC-3'
	R: 5'-GATGCAATTGTCTTCTACTGGTTC-3'
IL-1β	F: 5'-GCACGATGCACCTGTACGAT-3'
	R: 5'-AGACATCACCAAGCTTTTTGCT-3'
CRP	F: 5'-GTCTGCTACGGGGATTGTAGA-3'
	R: 5'-CACCGCCATACGAGTCCTG-3'
TGF-β	F: 5'-TCCCCCGAGAGGCAGATC-3'
	R: 5'-ATCGAGATGAGCGCTCTCTGA-3'
INF-γ	F: 5'-GGAACCCTCTCCCTTCAATGT-3'
	R: 5'-CTCCACAATAGCCTTCAGTGC-3'
CYP1A1	F: 5'-CCTCTTTGGAGCTGGGTTT-3'
	R: 5'-AGGCTCCACGAGATAGCAGT-3'
HSP70	F: 5'-CAGACGCAGACCTTCACTAC-3'
	R: 5'-TTTTGTCCTGCTCGCTAATC-3'
Nrf2	F: 5'-CTTTTGGCGCAGACATTCC-3'
	R: 5'-AAGACTGGGCTCTCGATGTG-3'
Keap1	F: 5'-CAACTTCGCTGAGCAGATTGGC-3'
	R: 5'-TGATGAGGGTCACCAGTTGGCA-3'
HO-1	F: 5'-CGCCTTCCTGCTCAACATT-3'
	R: 5'-TGTGTTCCTCTGTCAGCATCAC-3'
GCLC	F: 5'-GGAGGAGAGAGAGGCCTGGA-3'
	R: 5'-ATCGATGGTCAGGTCGATGT-3'
GST	F: 5'-GCTCTTACCACGTGCAGCTT-3'
	R: 5'-GGCTGGGAAGAGGAAATGGA-3'
β-actin	F: 5'-GCTCCTCCTGTTCGACAGTCA-3'
	R: 5'-ACCTTCCATGGTGTCTGA-3'

NF-kB, nuclear transcription factor-kB; TNF-α, tumor necrosis factor α; MIF, macrophage migration inhibitory factor; IL-2, interleukin-2; CRP, C-reactive protein; TGF-β, transforming growth factor; INF-γ, interferon; CYP 1A1, cytochrome P450 1A; HSP70, heat shock protein 70; Keap1, Kelch-like ECH-associated protein 1; HO-1, heme oxygenase; GCLC, glutamate cysteine ligase catalytic subunit; and GST, glutathione S-transferase.

### Enzyme-linked immunosorbent assay (ELISA)

The kidney tissues homogenate was obtained, followed by centrifugation for 15 min at 4°C. Next, the supernatant was detected by commercial ELISA kit (Boster, Wuhan, China) for the contents of IL-1β, IL-2, IL-4, IL-18, IL-10, IL-8, IL-6, INF-γ, TGF-β, CRP, MIF, TNF-α, NF-kB, CYP1A1, HSP70, Nrf2, Keap1, HO-1, GCLC and GST as described by the manufacturer’s instructions.

### Statistical analysis

Data were presented as the mean ± SD. One-way ANOVA followed by multiple comparison was used for data comparisons based on SPSS software. P < 0.05 was considered significant.
